# Extending Ethnoprimatology: Human–Alloprimate Relationships in Managed Settings

**DOI:** 10.1007/s10764-017-0006-6

**Published:** 2017-12-21

**Authors:** Alexandra Palmer, Nicholas Malone

**Affiliations:** 1 UCL Anthropology, University College London, WC1H 0BW, London, UK; 2 Anthropology, School of Social Sciences, University of Auckland, Auckland, 1010 New Zealand

**Keywords:** Conservation, Ethnoprimatology, Human–primate interfaces, Rehabilitation and reintroduction, Sanctuaries, Zoos

## Abstract

The majority of studies in ethnoprimatology focus on areas of sympatry where humans and nonhuman primates (hereafter, primates) naturally coexist. We argue that much can be gained by extending the field’s scope to incorporate settings where humans manage most aspects of primates’ lives, such as zoos, laboratories, sanctuaries, and rehabilitation centers (hereafter, managed settings). We suggest that the mixed-methods approach of ethnoprimatology, which facilitates examination of both humans’ and primates’ responses to one another, can reveal not only how humans’ ideas about primates shape management strategies, but also how those management strategies affect primates’ lives. Furthermore, we note that a greater focus on managed settings will strengthen links between ethnoprimatology and primate rights/welfare approaches, and will introduce new questions into discussions of ethics in primatology. For example, managed settings raise questions about when it might be justifiable to restrict primates’ freedom for a “greater good,” and the desirability of making primates’ lives more “natural” even if this would decrease their well-being. Finally, we propose that because ethnoprimatology is premised on challenging false dichotomies between categories of field site—specifically, between “natural” and “unnatural” free-ranging populations—it makes sense for ethnoprimatologists to examine settings in which humans exert considerable control over primates’ lives, given that the distinction between “wild” and “captive” is similarly unclear.

## Introduction

Ethnoprimatology questions the existence, and prioritization, of “natural” primate populations: owing to the increasingly anthropogenic context in which many primate populations live, ethnoprimatologists argue that human–alloprimate interactions are important for understanding primate behavior, ecology, and evolution (Fuentes 2012; Riley 2013; Riley *et al.* 2017). To study human–alloprimate interfaces, ethnoprimatologists draw not only on ethology, the traditional tool of primate field studies, but also on methods such as ethnography, interviews, surveys, and historical and economic analyses (Dore *et al.* 2017; Fuentes 2012). This methodological and theoretical hybridity allows ethnoprimatologists to examine how humans and primates respond to one another, rather than focusing on one side of the interaction. Following Sponsel (1997), who developed ethnoprimatology as a way of examining the breadth of the human niche in Amazonia, studies in ethnoprimatology have largely focused on zones of sympatry, where humans and nonhuman primates are entangled both socially and ecologically (Fuentes and Hockings 2010; Malone *et al.* 2014b; Riley *et al.* 2011). Accordingly, niche coconstruction—the mutual building and modification of human and primate ecological niches—has served as a core theoretical concept for investigating these settings (Fuentes 2012; Fuentes and Baynes-Rock 2017). In this article we argue for a deliberate extension of ethnoprimatology to incorporate settings where humans manage most aspects of primates’ lives, such as zoos, laboratories, sanctuaries, and rehabilitation centers, hereafter “managed settings.”

Our objectives in this article are, first, to explore the advantages of using mixed qualitative and quantitative methods, as employed in ethnoprimatology, for examining how human management practices and primate behavior mutually shape each other. Second, we consider the new kinds of ethical questions that would be introduced into ethnoprimatology, and hence primatology more broadly, as a result of further engagement with animal rights and welfare approaches, which historically have focused on animals in managed settings rather than those in free-ranging contexts. We finally explore how, because there is no coherent distinction between “captive” and “wild” environments, examining managed settings is a logical extension for ethnoprimatology, which is committed to questioning the existence of “pristine” field sites.

A few studies of human–alloprimate relationships in managed settings might be considered ethnoprimatology, though far more research on the subject has originated from other disciplines. Two studies by primatologists came out in the early 2000s that examined human–alloprimate interactions in managed settings. While Malone *et al.* (2000) used ethological methods to examine how human caregivers featured in postconflict behavior among chimpanzees (*Pan troglodytes*)—e.g., as neutral, affiliative partners or as safe outlets for redirected aggression—at the Chimpanzee Human Communication Institute (CHCI) in Ellensburg, Washington Baeckler (2001) used both ethological and ethnographic methods to examine how organizational cultures affected chimpanzee responses to caregivers at the CHCI and at two US zoos. More recently, A. Palmer conducted a study similar to Baeckler’s, using ethological and ethnographic methods to study relationships between orangutans (*Pongo pygmaeus, P. pygmaeus × abelii*) and keepers at Auckland Zoo, New Zealand (Palmer 2012; Palmer *et al.* 2015, 2016).

Because chimpanzees’ well-being was a central focus for both Malone *et al.* and Baeckler, these two studies share the goals of a larger body of work concerned with examining how human–animal relationships affect animal well-being in managed settings (Hosey 2008). With primates, ethological research on human–animal relationships has frequently suggested positive effects on primate behavior, such as increased affiliative behavior with conspecifics and reduced aggression and abnormal behavior, deriving from interactions with familiar caregivers (Baker 2004; Manciocco *et al.* 2009; Pizzutto *et al.* 2007) or from familiar caregivers’ use of species-typical behaviors and vocalizations (Jensvold 2008; Jensvold *et al.* 2010). Interactions with caregivers are not always associated with indicators of positive welfare though, such as one case when chimpanzees displayed more agonism and less prosocial behavior when caregivers were present (Chelluri *et al.* 2013). Interactions with unfamiliar humans such as zoo visitors are often positively correlated with behavioral indicators of stress (Birke 2002; Blaney and Walls 2004; Chamove *et al.* 1988), which may reflect a fundamental distinction between the effects of familiar and unfamiliar humans on primate welfare (Smith 2014). However, because the presence of unfamiliar humans is not consistently associated with negative welfare indicators (Bloomfield *et al.* 2015; Choo *et al.* 2011), individual histories of relationships with humans may play an important role in shaping animals’ responses to unfamiliar humans (Hosey 2008).

Although welfare-oriented research on human–primate relationships in managed settings occasionally involves limited use of interviews (Waitt *et al*. 2002) or questionnaires (Hosey and Melfi 2012), it tends to rely exclusively on ethological methods. Thus, unlike research in ethnoprimatology, the human side of the interaction tends to be omitted. In contrast, research in social anthropology and related disciplines has historically tended to focus on the role of animals, including alloprimates, in human social life: animals have been used as “a vehicle with which to explore a particular social formation or process” rather than as agents whose actions in turn shape human social worlds (Mullin 2002, p. 388). Increasingly though, social anthropologists are paying attention to human “entanglements” with animal agents (Kohn 2007, p. 4), on the grounds that animals are not just “good to think” with (Lévi-Strauss 1963) but are “here to live with” (Haraway 2003, p. 5). Thus, multispecies ethnography research considers how other living organisms—including mushrooms (Tsing 2012) and entire forests (Kohn 2013)—“shape and are shaped by political, economic, and cultural forces” in human societies (Kirksey and Helmreich 2010, p. 545). Whereas multispecies ethnographers tend to employ qualitative methods, primarily ethnography, for examining human–animal relationships, “zooethnography” incorporates methods from animal behavioral science and so resembles ethnoprimatology when human–alloprimate relationships are the subject (Parreñas 2012). For example, Parreñas (2012) used focal animal sampling, participant observation and interviews, and archival research to explore relationships between humans and orangutans (*Pongo pygmaeus*) at a rehabilitation center in Sarawak, Malaysia, paying attention to both intimate encounters between caregivers and orangutans and the effects of ethnic and gender hierarchies on human and orangutan lives. Other research from social anthropology and related disciplines has examined human–primate relationships, and political and discursive contexts, in laboratories (Arluke 1992; Arluke and Sanders 1996; Birke 2007; Birke and Arluke 2006; Coleman 2011; Sharp 2015) and ex situ sanctuaries (Alcayna-Stevens 2012; Hua and Ahuja 2013).

Furthermore, there is a substantial literature in fields such as history, philosophy, and literature and media studies focusing on representations of primates, including those in managed settings. For example, Cribb *et al.* (2014) trace the history of orangutans in Western culture, including as subjects of research, conservation, and rights discourses, and as figures represented in the arts, zoos, and taxidermy displays. Haraway (1989) more broadly considered the role of primates in Western science—including field primatology, laboratory-based research, museums, and even space exploration—paying particular attention to how ideas about race and gender shape media representations and scientific theory and practice. Corbey (2005) specifically focuses on the representation of apes within Western discourses, especially those contributing to anthropology, emphasizing how primates have been cast as liminal creatures marking the boundary between humans and animals; contributions to a symposium organized by Corbey and Theunissen (1995) additionally consider representations of apes in non-Western cultures. The ethics and symbolism of keeping primates in zoos and laboratories have also received considerable attention from philosophers, as we will discuss later in the article.

To support our argument for including managed settings in ethnoprimatology, we draw on literature from human–animal relationship research, multispecies ethnography, and humanities disciplines, as well as our own research with apes in zoos and rehabilitation centers. A. Palmer has conducted two research projects on humans’ relationships with orangutans in managed settings, the first of which was conducted at Auckland Zoo, New Zealand, in 2012, and involved ethnographic research with the six orangutan keepers, and 102 h of behavioral observations of four of the six orangutans housed at the zoo, using a combination of instantaneous scans, event frequencies, and one-zero sampling (Altmann 1974; see Palmer 2012; Palmer *et al.* 2015, 2016). Since 2015 A. Palmer has conducted research on debates about ethics and philosophy in orangutan rehabilitation. This research has involved in-depth semistructured interviews, written correspondence, and informal conversations with, to date, more than 90 practitioners, most of whom have some decision-making capacity in orangutan conservation (including donors, researchers, knowledgeable commentators, and managers at rehabilitation centers, field projects, and head offices), and visits to the majority of orangutan rehabilitation projects in Indonesia and Malaysia on the islands of Borneo and Sumatra. N. Malone works collaboratively on applied research that is designed to inform and calibrate conservation interventions with both free-living and displaced silvery gibbons (*Hylobates moloch*) in West Java (Malone *et al.* 2017, 2014a, b). In this research, N. Malone and colleagues combine ethological observations (>300 h of scan sampling and fixed-point counts of gibbon vocalizations), ecological monitoring (longitudinal assessments of habitat alterations over a 10-yr. period from 2005 to 2015), and ethnographic methods (participant observation, cultural mapping, and semistructured interviews with more than 50 participants) in various research settings including nature reserves, villages, and rehabilitation centers.

### Advantages of Studying both Sides of Human–Primate Interactions in Managed Settings

In managed settings, using a combination of quantitative and qualitative methods can help explore how humans’ cultural and individual ideas about primates affect management practices, and how management practices in turn affect primate behavior and welfare. For example, as we have discussed in greater depth elsewhere (Palmer *et al.* 2015), A. Palmer’s research at Auckland Zoo demonstrated that conflict between females featured prominently in keepers’ accounts of why specific husbandry practices had been selected, such as feeding females in separate areas. However, A. Palmer’s ethological observations revealed few instances of female–female conflict. In particular, interactions between Melur and Wanita, two females whose relationship was a source of concern for keepers, were relatively rare, with A. Palmer observing three cases when Wanita took Melur’s food (and several unsuccessful attempts halted by keepers’ interventions), one displacement event, and one fight lasting ca. 20 s following Melur’s attempt to take some of Wanita’s food. Keepers also reported one “little spat” between the females, two displacement events, and two cases when Melur seemed “wary” of Wanita during the research period. However, the females’ interactions—which were the least common of all dyads, with the females spending on average 8.11% of 5-min scans per 1-h sample within 2 m—were usually characterized by tolerance rather than aggression. For example, A. Palmer observed 11 occasions when the two females remained in close proximity while alternately playing and resting with Madju (Melur’s son). Furthermore, analysis of each female’s approaches to and withdrawals from the other revealed that Melur, whom keepers believed to be fearful of Wanita, was more responsible for maintaining proximity than Wanita: the pair’s Hinde’s Index was 26.31, on a scale of −100 to 100 (Hinde and Atkinson 1970). Keepers could therefore be seen as hypervigilant of female–female aggression, which we attributed primarily to the history of conflict between females at the zoo and to keepers’ view that preventing distress and injury is a central goal of zookeeping. Furthermore, keepers emphasized how aggressive interactions between orangutans are subtle, far more so than among chimpanzees, so many behaviors that do not appear clearly aggressive can be, in the words of one keeper, “quite a big statement” for an orangutan, such as one female moving away as the other moves in her direction. As such, it is possible that keepers were far more perceptive than A. Palmer at detecting aggressive interactions.

The aforementioned research illustrates how combining quantitative ethological methods to observe primate behavior and qualitative methods such as interviews and participant observation to gather humans’ perspectives can facilitate understanding of how interpretations of primate behavior shape management practices. Using such mixed methods can also reveal how those management choices affect primate behavior and welfare. For example, Baeckler (2001) found that differences in husbandry practices between two zoos and the CHCI related, among other things, to caregivers’ ideas about how much respect and autonomy chimpanzees deserve. At two study sites caregivers spoke explicitly about how certain practices, such as not putting their body parts into the chimpanzees’ cages, reflected their respect for the chimpanzees, whereas at the third site the primary keeper described wanting to “get respect out of” the chimpanzees, which he described as “my animals” (p. 91). Through interviews with keepers and ethological observations of human–chimpanzee interactions, Baeckler demonstrated that the facility where the keeper paid least attention to treating chimpanzees in a respectful manner also had the lowest frequencies of cross-species affinitive social behaviors and play and the highest frequency of cross-species threat behavior, and chimpanzees were least likely to cooperate with the keeper’s instructions or requests. As such, Baeckler concluded that differences between management “cultures” at the three sites affect the quality of relationships between chimpanzees and caregivers, which in turn affect intraspecific social interactions. Baeckler noted that an appropriate extension of this research would involve observing chimpanzees in the absence of caregivers to test whether interactions with caregivers affect chimpanzees’ overall behavior and well-being.

A mixed methods approach to studying human–alloprimate relationships in rehabilitation contexts could reveal similar connections between interpretations of primate behavior, and primate behavior and well-being. A. Palmer’s current research on orangutan rehabilitation illustrates how ideas about orangutan behavior can affect how different institutions carry out the process of rehabilitation—which involves helping animals to become healthy, independent of humans, and socially and ecologically capable of surviving with greater independence in the wild (Beck *et al.* 2007). For example, the level and kind of contact between infant orangutans and “babysitters” advocated by rehabilitation center managers depend on managers’ views about how much contact with humans is helpful for orphaned orangutans’ psychological development. While some stress the importance of providing a surrogate mother for healthy development, others suggest that the kinds of bonds that young orangutans form with human caregivers are unhealthy, fostering undesirable human orientation later in life, or could lead to further trauma when youngsters are separated from their caregivers (FORINA 2013; Rosen and Buyers 2002; Russon 2009; Russon *et al.* 2016). Furthermore, the extent to which rehabilitation managers actively discourage ongoing relationships with caregivers as orangutans approach the point of release depends, in part, on the extent to which they view orangutans’ “semisolitary” existence as innate. For example, one practitioner argued that because orangutans tend to become solitary as they age as part of a “natural process,” few measures are required to discourage ongoing “humanization” and attachment to caregivers. In contrast, another organization undertakes a staged process in which bonds between infants and their (female) caregivers are encouraged, but contact between older juveniles and adolescents and their (male) caretakers is strongly discouraged, demonstrating a perception that “dehumanization” must be actively fostered in the rehabilitation process (Russon *et al.* 2016).

Similarly, in silvery gibbon rehabilitation, ideas about the gibbons’ “natural” social system affect management strategies and outcomes. While the development of stable pair bonds between adult males and females is often prioritized (among other criteria, such as exhibiting effective locomotor and foraging behavior) in the prerelease context, recent evidence suggests that pair splitting is common after release (Smith 2011; Wedana and Jeffery 2016). In light of challenges to the primacy of the two-adult group and monogamous mating patterns within hylobatid social systems, conservation managers are faced with the challenge of overcoming traditional (and often culturally mediated) characterizations of “preferred social units,” as well as constraints with respect to providing opportunities for the types of social and ecological interactions with conspecifics that likely underpin species-typical patterns of dispersal, reproduction, and inter- and intragroup relationships (Fuentes 2000; Malone and Fuentes 2009).

Further research combining ethological observations of rehabilitant primates and the views of rehabilitation managers would provide additional detail on how culturally mediated ideas, such as the innateness of orangutans’ solitariness and gibbons’ monogamous mating, affect primate behavior during and after rehabilitation. More broadly, ethnoprimatologists’ use of mixed methods to focus on both human and primate sides of the interaction could greatly enrich our understanding of interspecific relationships in managed settings, through revealing how humans’ ideas about primates and their behavior shape management strategies, and how those strategies in turn affect primate behavior and well-being.

## Ethics and Managed Settings

A second contribution of ethnoprimatology in managed settings would be to reinforce the connections between ethnoprimatology, and hence primatology more broadly, and primate welfare/rights approaches. As Fuentes (2012) explains, critics have suggested that primatologists tend to downplay primate agency and histories of exploitation and oppression, thereby necessitating further engagement with primate rights and welfare discourses. Although there has been some engagement with primate rights and welfare within primatology, as demonstrated by some primatologists’ support for the Great Ape Project (GAP) (Beck *et al.* 2001; Cavalieri and Singer 1993; Corbey and Lanjouw 2013) and discussions of empathy and other facets of primatologists’ relationships with research subjects (Vitale and Pollo 2011), Fuentes notes that there has been little academic engagement with the rights and welfare of captive primates in the West. This lack of engagement is perhaps because, as Beausoleil (2014) points out, animal welfare and rights approaches have rarely focused on “wild” animals—for example, Singer (1975) and Regan (1983) mention wild animals only briefly in their seminal texts (Hargrove 1992a). Not only, Beausoleil argues, is it impractical to prevent discomforts that are part of natural processes (one could not realistically ensure that all members of a wild species receive adequate food), but also advocates of animal welfare and rights often prioritize autonomy over well-being. For example, Bekoff (in comments on Jamieson 1995a, p. 47), a prominent supporter of animal welfare and rights, argues that although “it is too bad that deer get killed by wolves” and he “would perhaps like the world to be different,” because “there is a sense of wildness that is beautiful, that we should respect and admire,” wildness should not be sacrificed for the sake of welfare. The centrality of autonomy also underpins the goal, proposed by some advocates of animal rights and liberation, of abolishing institutions involving humans’ exploitation of captive animals, such as animal testing laboratories, zoos, and industrial agriculture (Jamieson 1985; Kopnina 2017; Regan 1983; Singer 1975).

Although the focus of animal rights and welfare approaches has historically been on captive animals, some attempts have been made recently to engage with wild animals. For example, advocates of “compassionate conservation” seek to make concern for individual animals’ suffering central to conservation (Aitken 2004; Beausoleil 2014; Bekoff 2013, 2014; Paquet and Darimont 2010; Ramp and Bekoff 2015), as a way of unifying animal rights/welfare and conservation approaches, which sometimes disagree on the ethics of killing or harming individual animals for a conservation goal (Hargrove 1992b), such as killing “pest” (Ben-Ami *et al.* 2014; Nagy and Johnson 2013; Ramp 2013) or “surplus” (Lindburg 1991) animals. Compassionate conservation approaches have created some opportunities for ethnoprimatologists to engage with animal rights/welfare, such as through examining bushmeat hunting of great apes and other primates in equatorial Africa (Bekoff 2013; Rose 2011). Still, the kinds of ethical issues that are raised in free-ranging contexts (MacClancy and Fuentes 2013; MacKinnon and Riley 2010)—such as the ethics of hunting endangered primates (Wadley *et al.* 1997), habituation (Goldsmith 2014), and long-term research presence (Strier 2010)—are different from those raised in managed settings, so focusing on managed settings offers an opportunity for reflection on a new set of ethical concerns. For example, Western countries rarely have groups of free-ranging primates, yet frequently have managed settings such as zoos and laboratories. This means that managed settings present a unique opportunity to examine the kinds of human–primate relationships, and ethical considerations, that emerge among people who lack the experience of living alongside free-ranging primates. Because a significant proportion of primatologists are also from Western nations, especially North America (Fuentes 2011), examining human–alloprimate relationships in the West might present an opportunity to reflect on cultural ideas present within Euro-American primatology. Furthermore, each kind of managed setting presents a unique set of ethical considerations with which ethnoprimatologists might engage.

Ethical issues in zoos relate to the acceptability of restricting the freedom of animals for the sake of public education or breeding “insurance populations” in case of extinction in the wild, which are the two most common justifications for the existence of modern zoos (Bulbeck 2005; Rothfels 2002). As well as questioning the legitimacy of “ark” narratives (Stanley Price and Fa 2007) and educational efficacy (Balmford *et al.* 2007), critics have expressed concern about animal welfare, particularly for highly intelligent animals such as great apes. Despite efforts of zoos to enhance animal welfare, encourage “natural behavior” (Bostock 1993; Bracke and Hopster 2006), and use naturalistic enclosure design to make exhibits appear more comfortable than the “real ‘wild’” (Rothfels 2002, p. 216), “abnormal” behaviors such as self-injurious and stereotypic behaviors remain prevalent in captive primates. Though it is possible that the performance of abnormal behaviors helps alleviate suffering by replacing an absent behavior that would be performed in the wild (Mason and Latham 2004), and in general understanding the psychological motivations of animals’ behavior presents a challenge (Mitchell *et al*. 1997), abnormal behaviors are generally regarded as indicating deprived environments (Br*ü*ne *et al.* 2006). Even primates in accredited zoos display abnormal behaviors (Birkett and Newton-Fisher 2011; McComb 2009), which Birkett and McGrew (2013, p. 142) argue means that “the only defensible reason for [keeping apes in captivity] is to offer lifelong sanctuary to those who cannot be returned to nature.” From a rights perspective, the principles of encouraging animals’ autonomy and refraining from using animals for humans’ benefit led Jamieson (1995b, p. 62) to reject the notion that having animals in a zoo is better than not having them at all, as that would simply “transform animals into exhibits in a living museum.” Critics have also employed semiotic analyses to question the power dynamics and educational messages of the zoo, arguing that the zoo establishes a power dynamic between observed and observer, which reinforces dominance-based attitudes to wildlife and thereby “debases the watcher as well as the watched” (Malamud 1998, p. 5; see also Acampora 2005; Berger 1980).

A. Palmer’s research at Auckland Zoo considered keepers’ perspectives on the ethics of compromising orangutan welfare and autonomy in the zoo (Palmer 2012; Palmer *et al*. 2016). Keepers felt conflicted by their roles, as they spoke of orangutans as “people” and sometimes explicitly voiced support for great ape rights. Keeping orangutans as “prisoners” was therefore, as one keeper put it, a “gray area” and a “tricky issue,” since “they’re almost a human in a cage.” Two of the six keepers found it particularly difficult to justify their roles, with one describing feeling “guilty, all the time” and the other explaining it “breaks our hearts” to see orangutans in cages. Yet keepers were ultimately able to justify their roles to themselves by either pragmatic reasoning (“They’re here. We can’t do anything about that realistically, so who better to look after them than me, or us, people who really, really care?”), or by invoking educational or ark justifications for zoos.

Laboratories offer a slightly different, though related, set of ethical questions. First, whereas zoo animals might be construed as individuals whose freedom has been compromised for the sake of their species, laboratory animals are used for the good of humans (Malone *et al.* 2010), rendering animal testing incompatible with some formulations of animal rights (Regan 1983). From an animal welfare perspective, abnormal behaviors and other symptoms of psychological distress can be improved by positive interactions with laboratory caregivers, but such interactions can take an emotional toll on caregivers as they struggle to kill and harm valued social partners (Coleman 2011). As such, Arluke (1992; Arluke and Sanders 1996) found that caregivers at one laboratory, who were generally self-described “animal lovers,” sought to provide optimal welfare for their primate charges but struggled to justify their roles ethically. One way that caregivers sought to remedy their ethical conflict was to view laboratory primates as “sacrifices,” in some cases performing rituals such as a minute of silence or prayers before euthanizing a primate (if “euthanasia” is the correct term: Broom 2007; Morris 2012; Rowan 1995). However, speaking about emotional difficulties and ethical dilemmas largely remained “taboo,” which Birke (2007) links to feminist theory, arguing that “masculine” laboratory cultures lead to a focus on species hierarchies and the suppression of emotions. Furthermore, caregivers at a second laboratory examined by Arluke tended to exhibit less care for their charges, which concurs with other research showing that laboratory scientists deindividualized their charges, treating laboratory animals as a separate category of creatures whose “sole purpose in life was to serve in a scientific experiment” (Phillips 1993, p. 76, 1994).

In addition to the ethics of using other animals for the benefit of human health, laboratories raise questions about prioritization of highly intelligent species, such as primates, over those lower on the “Great Chain of Being.” The close evolutionary relationship between humans and other primates, especially chimpanzees, has been used to justify the use of primate models in biomedical testing; however, their value as human models is limited, such that the Institute of Medicine (2011) has declared that “most current use of chimpanzees for biomedical research is unneccessary” (Ross 2014). Furthermore, great apes’ similarity to humans, advanced cognitive abilities, and rich emotional lives might grant them an “unusually strong claim to high status” in moral considerations (Warren 2001, p. 318). For this reason, the Great Ape Project aims to eliminate the use of great apes in laboratories (Cavalieri and Singer 1993; GAP 2017). In recent years the move to eliminate research with great apes has accelerated, with the US National Institutes of Health committing to phase out research with chimpanzees (NIH 2015).

Chimpanzees “retired” from medical research, and many primates displaced by habitat destruction or caught up in illegal trade, are sent to sanctuaries, where they are ideally provided with good welfare until death (Ross 2014; Trayford and Farmer 2012). Ethical issues raised by sanctuaries include the contradiction that while attempting to provide sanctuary primates with a “good life,” keepers simultaneously await and even welcome their charges’ deaths (Hua and Ahuja 2013). Furthermore, sanctuaries are captive settings that are, paradoxically, justified by anticaptivity discourses such as animal rights and criticism of biomedical testing on animals. By “accepting the reality of lifelong captivity,” the sanctuary thereby “reveals limits to animal rights discourses” (Hua and Ahuja 2013, p. 627). As in zoos, sanctuaries may aim to improve primate welfare through encouraging “natural behavior,” which relies on the notion that respectful treatment involves minimizing human influence (or “dehumanizing” primates), despite the presence of social and emotional bonds between keepers and their charges (Alcayna-Stevens 2012).

The priority given to providing “natural” environments is one justification for choosing rehabilitation and reintroduction over sanctuary care. Some view reintroduction projects, such as sanctuaries, as primarily justified by their conservation value, such as enabling enforcement of wildlife trade and hunting laws through confiscation of illegal pets (Rijksen and Meijaard 1999) or providing other benefits such as community education (Teleki 2001). However, data on postrelease survival or reintroduced primates are often lacking, or show unsatisfactory survival rates (Cheyne 2009; Russon 2009; Russon *et al.* 2016), fueling the argument that habitat protection is a far more cost-effective conservation tool than reintroduction (Wilson *et al.* 2014). For others, the primary justification for reintroduction is its potential to increase animal welfare, though critics have questioned this argument on the grounds that release into the wild can be highly stressful and not necessarily conducive to good welfare (Humle and Farmer 2015; Moore *et al.* 2014).

Some tensions between the priorities of welfare and “wildness” became apparent in A. Palmer’s research on orangutan rehabilitation and reintroduction, in which practitioners disagreed as to which orangutans are “unreleasable” because of poor survival skills. On the one hand, some practitioners interviewed by A. Palmer indicated that they would rather release orangutans even if their survival skills are dubious. Several reasons were presented for this view, including the idea that the conservation benefits of reintroduction justify individuals’ suffering after release, and that orangutans fare poorly in captivity, especially in the inadequate cages in most rehabilitation centers (Trayford 2013). Some even indicated that they would rather see orangutans have “death with dignity” in the wild than live their whole lives in a cage (Seal 1991), indicating a preference for making primates’ lives as “natural” as possible, even if naturalism comes at the expense of primates’ well-being or survival. On the other hand, others emphasized the need to avoid “sending them out to die” and suggested that, even if the conservation element or public appeal suffered, it would be more ethically responsible to provide a good life in captivity for any orangutans that may not be able to survive independently. One practitioner argued that there is a degree of “freedom arrogance” among orangutan rehabilitation and reintroduction practitioners, as it is often assumed that life in the wild is always better than life in a cage. Similar conflicts between priorities of welfare and wildness emerged in N. Malone’s research on gibbon rehabilitation, in which practitioners debated whether to prioritize the welfare of released gibbons or the “naturalness” of the release site conditions. For example, apex predators such as leopards (*Panthera pardus*) at release sites were flexibly described as both “indicators of a suitable release site” and “an operational liability” given the naiveté of gibbon release candidates. The latter characterization was then contextualized as being preferable to the presence of human hunters, reflecting the idea that a “natural” death from leopard predation was preferable to death at the (“unnatural”) hands of humans.

As these examples illustrate, the kinds of ethical questions raised within managed settings differ from those raised within wild contexts, and offer opportunities for building stronger connections between primatology and animal rights/welfare discourses. Among other matters, zoos, laboratories, sanctuaries, and rehabilitation centers raise questions around the circumstances under which primates’ autonomy might be sacrificed for the “greater good” (of their own species, or the good of humans), the emotional challenges faced by keepers who might struggle to justify their roles, and the desirability of preserving “naturalness” or “wildness” even if this comes at the expense of primate well-being or survival.

## Beyond the Captive–Wild Dichotomy

Questioning the existence, and prioritization, of “natural” primate populations unaffected by human presence is a fundamental premise of ethnoprimatology (Fuentes 2012; Riley 2013; Riley *et al.* 2017). Just as the dichotomy between “natural” and “unnatural” free-ranging primate populations is false, following Braverman (2014, 2015), we suggest that there is no clear divide between “wild” and “captive.” We propose that it therefore makes sense for ethnoprimatologists to consider human–primate relationships at all kinds of sites, not simply areas of sympatry where humans’ and primates’ ranges overlap.

The first problem with coherently distinguishing “wild” and “captive” primate populations is that there is a continuum in the level of influence humans have over primate groups, not a dichotomy between those free from human effects and those completely controlled by humans. As Braverman (2014, p. 54) points out, there is a “growing array of gray sites” where animal populations are not obviously “natural” or “captive,” such as game reserves in Africa in which animals are intensively managed but remain in their original locations. We acknowledge that there is no single continuum of human influence on primates, but rather many dimensions to human–primate relationships. For example, there is variation in the degree to which management of primates is formally controlled, the mechanisms and reasons for management, and the ways in which primates exert their own influence on human lives. However, our focus is specifically on the degree to which human activities influence primate social and ecological behavior, as this is the central factor underpinning common definitions of “wild” and “captive.” As many authors have discussed, Western worldviews are often characterized as regarding humans and the realm of “culture” as fundamentally separate from “nature,” and are held in contrast to “societies of nature,” which view humans as a part of nature (Cronon 1996; Descola and Pálsson 1996; Dwyer 1996; Posey 2004). Though reality is clearly more complex (e.g., Roman Catholic ideas about nature are quite diverse: Binde 2001), Aitken (2004, p. 58) argues that the view of humans as separate from nature remains fundamental to “mainstream” conservation, since “if we are nothing but nature, then whatever action we take must be the right one” (e.g., an oil spill would be seen as a perfectly natural change to an ecosystem). Similarly, Braverman (2014, 2015) argues that the preservation of “natural,” or “in situ,” ecosystems remains the top priority for most conservationists. Though strains of conservation philosophy incorporate humans into the realm of nature—e.g., “new conservation” approaches explicitly make room for local peoples’ use of their surrounding environments (Caro *et al.* 2012; Hunter *et al.* 2014; Kareiva 2014; Marris 2014; Marvier 2014; Miller *et al.* 2014; Soulé 2013)—the view of humans as separate from nature remains prominent, and underpins common distinctions between “wild” and “captive” animals (Aitken 2004; Braverman 2014, 2015). We therefore focus on a continuum in the degree to which humans influence primate behavior for heuristic purposes.

At one end of the spectrum of human influence, “wild” populations of primates are hypothetically unaffected by anthropogenic activities. However, ethnoprimatologists have pointed out that few, if any, primate populations live in such a state, since usually primates’ lives have been affected by present or past anthropogenic activities, which might relate to landscape or dietary modification, hunting, or habituation to or harassment from people (locals, tourists, or researchers: McKinney 2015). In these settings, human effects on primate behavior are in many cases unintentional (e.g., habitat modification is usually not specifically intended to change primate behavior), though in other cases humans deliberately set out to change primate behavioral patterns, such as when researchers seek to habituate subjects (Hanson 2017). In more anthropogenic environments such as urban areas and agricultural settings, humans might again unintentionally modify primate behavior—e.g., altering primate diets by introducing crops—or might seek to more explicitly manage primate behavior, e.g., by driving away crop-feeding primates (Lee 2010) or attracting primates with provisions, as at Hindu temples (Fuentes 2010; Solomon 2016). Further along the management continuum, tourism sites where primates are provisioned, such as Japanese monkey parks, reflect an even greater degree of management of primates’ lives where nutrition, and consequently ranging patterns, are deliberately modified (Knight 2005). At the extreme end of managed settings are zoos and laboratories, where almost every aspect of primates’ lives is managed by humans (Hosey 2005).

A further issue with distinguishing “wild” and “captive” primate populations is that it is not possible to place a specific category of site—e.g., a zoo, a rehabilitation center, or a rural sympatric environment—in a consistent location along the continuum, since there is substantial variation within, and between, categories. For example, rehabilitation centers, where primates are housed while they undertake the process of rehabilitation, typically resemble settings such as zoos insofar as humans control primate nutrition, social interactions, housing, and breeding. However, there is no clear distinction between the “captive” rehabilitation center and the “wild” reintroduction site, where primates are finally released once rehabilitation is complete. This is, first, because rehabilitation and reintroduction projects often deliberately decrease the level of human management as primates age, with the idea of making the transition from captivity to the wild gradual. For example, three orangutan rehabilitation centers—Nyaru Menteng (BOS Foundation 2016), International Animal Rescue (2015), and the Centre for Orangutan Protection—house release candidates on natural or artificial islands, where space is still restricted, food provided, and reproduction prevented, but orangutans experience considerable independence. Furthermore, the act of release is by no means the end of human management, as there is always some degree of intervention at release sites. At orangutan reintroduction sites, some projects continue to provide supplemental food for released orangutans throughout their lives, with the idea of reducing pressure on any wild populations in the area and offering orangutans “choice” should they wish to receive food from humans. Even those that, either gradually or abruptly, cease provisioning might continue to monitor orangutans’ progress after release and potentially intervene medically if released orangutans are in need. As illustrated by complaints from several orangutan reintroduction practitioners interviewed by A. Palmer that postrelease monitoring can compromise “wildness,” few released orangutans are truly free from human management, thereby blurring the boundary between “captive” rehabilitation centers and “wild” reintroduction sites.

There are also overlaps in terms of purpose and practice between kinds of sites, further blurring distinctions between categories. For example, orangutan rehabilitation centers and release sites that are open to tourists (e.g., Bukit Lawang, Tanjung Puting, Sepilok, Matang, and Semmengoh) resemble zoos in that they aim to educate visitors, expose primates to large numbers of unfamiliar humans, and continue to manage primates’ nutrition and even breeding throughout their lives. Breeding policies hypothetically distinguish zoos and rehabilitation centers: while many rehabilitation centers and sanctuaries prohibit breeding for welfare and management reasons (GFAS 2013; FORINA 2013; Russon 2009), accredited zoos carefully manage the genetics of captive populations through breeding exchanges (Stanley Price and Fa 2007). However, as both A. Palmer and Parre*ñ*as (2012, 2016) found, some rehabilitation and reintroduction projects with visitors actively encourage breeding, indicating that a key distinction between zoos and rehabilitation centers does not consistently hold true.

A final reason to challenge distinctions between captive and wild environments is that all primate populations are connected: physically through the movement of primates into and out of their original habitats, and conceptually in terms of the stated justifications for managed settings. As such, “in situ” and “ex situ” sites “affect each other in reciprocal ways” (Braverman 2014, p. 53).

For example, primates at rehabilitation centers were removed from free-ranging populations, and may be returned to the wild to either interbreed with existing populations or form new populations, though interbreeding with existing populations is controversial (Beck *et al.* 2007; FORINA 2013; Rijksen and Meijaard 1999; Rosen and Buyers 2002; Russon 2009; Smits *et al.* 1995). Historically, primates in zoos and laboratories were also removed from their original habitats, though today progressive zoos refrain from this practice and instead sustain captive populations through managed breeding programs. Rather, progressive zoos define their relationship to the wild quite differently, emphasizing not only their role as conservation educators but also as “arks” harboring threatened animals, some of whom may be required to repopulate the wild. Captive breeding in zoos followed by release has proved successful for some primates, most famously golden lion tamarins (*Leontopithecus rosalia*: Kleinman *et al.* 1991), and has recently been conducted with more mixed results with western gorillas (*Gorilla gorilla gorilla:* King and Courage 2008) and Sumatran orangutans (*Pongo abelii*: Bullo 2015; Cocks and Bullo 2008). Similarly, a female silvery gibbon named Regina—originally born in, and transferred between, Australian zoos before being sent to the United Kingdom—was recently “repatriated” to West Java (Wedana and Jeffery 2016).

The dichotomy between “captive” and “wild” environments is therefore, like that between “natural” and “unnatural” field sites, false. Not only is there a continuum in the degree of influence humans exert over primate populations, but also the level and kind of human influence vary within and between categories of sites. It is therefore impossible to, for example, state consistently how much, and what kind of, human control is exerted over reintroduced primates, or to coherently distinguish rehabilitation and reintroduction projects from zoos. In addition, there are connections between “wild” and “captive” primate populations in terms of physical movement between groups and stated justifications; for example, zoos are justified as maintaining an “ark” in case of animals’ extinction in the wild. For these reasons, we find it fitting that ethnoprimatology—a discipline founded on questioning false dichotomies between kinds of field sites—should explicitly focus on human–primate relationships in all kinds of settings, including those where humans control most aspects of primates’ lives.

## Conclusion

We have presented three central reasons why, we believe, extending ethnoprimatology’s scope to include managed settings would benefit ethnoprimatology itself and broader research on human–primate relationships in managed settings. The mixed-methods approach of ethnoprimatologists facilitates examination of both humans’ and primates’ responses to one another. In managed settings, this kind of approach can reveal not only how humans’ ideas about primates—derived from personal convictions, cultural ideas, and the individual- and species-level behavior of primates—shape management strategies, but also how those management strategies in turn affect primates’ lives. Second, because animal rights/welfare discourses primarily focus on animals in managed settings, incorporating managed settings into the scope of ethnoprimatology will strengthen the field’s links to welfare/rights approaches. Furthermore, the unique kinds of ethical questions raised by managed settings—such as the circumstances under which restricting primates’ freedom is justified for a “greater good”—will introduce new kinds of ethical questions into ethnoprimatology, and primatology more broadly. Finally, we propose that because ethnoprimatology is premised on challenging false dichotomies between categories of field site (specifically, “natural” and “unnatural” free-ranging populations), it makes sense for ethnoprimatologists to examine settings like zoos in which humans exert considerable control over primates’ lives, given that the distinction between “wild” and “captive” is similarly unclear.
